# IFNs in host defence and parasite immune evasion during *Toxoplasma gondii* infections

**DOI:** 10.3389/fimmu.2024.1356216

**Published:** 2024-02-07

**Authors:** Carsten G. K. Lüder

**Affiliations:** Institute for Medical Microbiology and Virology, University Medical Center Göttingen, Göttingen, Germany

**Keywords:** interferons, *Toxoplasma gondii*, immunity, immune evasion, host defence, signalling, parasite effectors

## Abstract

Interferons (IFNs) are a family of cytokines with diverse functions in host resistance to pathogens and in immune regulation. Type II IFN, i.e. IFN-γ, is widely recognized as a major mediator of resistance to intracellular pathogens, including the protozoan *Toxoplasma gondii*. More recently, IFN-α/β, i.e. type I IFNs, and IFN-λ (type III IFN) have been identified to also play important roles during *T. gondii* infections. This parasite is a widespread pathogen of humans and animals, and it is a model organism to study cell-mediated immune responses to intracellular infection. Its success depends, among other factors, on the ability to counteract the IFN system, both at the level of IFN-mediated gene expression and at the level of IFN-regulated effector molecules. Here, I review recent advances in our understanding of the molecular mechanisms underlying IFN-mediated host resistance and immune regulation during *T. gondii* infections. I also discuss those mechanisms that *T. gondii* has evolved to efficiently evade IFN-mediated immunity. Knowledge of these fascinating host-parasite interactions and their underlying signalling machineries is crucial for a deeper understanding of the pathogenesis of toxoplasmosis, and it might also identify potential targets of parasite-directed or host-directed supportive therapies to combat the parasite more effectively.

## 1 Introduction

*Toxoplasma gondii* is an obligatory intracellular parasite of birds and mammals including up to 30% of humans worldwide. After oral transmission, infectious sporozoites or bradyzoites infect the intestinal epithelium, transform into replicative tachyzoites and disseminate throughout the host’s body (1). Tachyzoites employ a lytic life style including active, parasite-driven host cell invasion, intracellular replication within a membrane-bound compartment called the parasitophorous vacuole (PV), egress and host cell lysis, and infection of new host cells (2). Importantly, *T. gondii* can infect virtually any nucleated host cell including immune and non-immune cell types. During acute infection, sentinels of the immune system including macrophages and dendritic cells (DCs) are preferred host cells which facilitate dissemination of the parasite (3–5). Such host cell range may critically influence the host response towards the parasite. Likewise, effectiveness and mechanisms of host immune responses clearly differ between host species, with differences being documented between mice, rats or humans (6). Recognition of *T. gondii* pathogen-associated molecular patterns (PAMPs) by pattern recognition receptors (PRRs) initiates an efficient, largely cell-mediated immune response that restricts parasite multiplication and kills the tachyzoite stage. However, concomitantly few parasites differentiate to largely dormant and non-replicating bradyzoites which form a rigid cyst wall and persist as intracellular tissue cysts mostly in neurons and muscle cells. Cellular immunity towards *T. gondii* is critical for restricting parasite propagation following primary infection and for avoidance of parasite reactivation of latent bradyzoites to tissue-destructing tachyzoites (7). Consequently, infection with *T. gondii* is regularly benign in immunocompetent hosts, but can be severe and life-threatening in immunocompromized patients or after intrauterine infection of fetuses from pregnant women with primary infection (1).

Interferons (IFNs) have been first described more than 60 years ago as molecules which “interfere” with virus replication (8). They are now known to comprise more than 20 family members including 13 IFN-α subtypes in humans. They fulfil various functions in immunity and inflammation but also in other cellular functions including growth, proliferation, differentiation, survival and motility (9). IFNs are classified into three classes, namely type I IFNs with IFN-α/β being the best charaterized members, a single type II IFN, i.e. IFN-γ, and four IFN-λ1-4 members in humans (two in mice). IFN-α/β are best known for their potent anti-viral effects in a broad range of cell types, but they can also combat intracellular bacteria and parasites (10). IFN-γ can induce potent anti-microbial effector mechanisms in bacteria- or parasite-infected hematopoietic and non-hematopoetic cells, and it is critical for triggering adaptive cellular immunity (11). IFN-λs primarily protect epithelial cells at mucosal surfaces during microbial exposure (12). All three classes of IFNs are induced during infections with *T. gondii*, and they play critical roles in anti-parasitic defence and regulation of immunity during toxoplasmosis. However, *T. gondii* has evolved several strategies to counteract IFN-mediated immunity, both at the level of host gene expression as well as at the level of individual effector mechanisms (13). The arms race between IFN-mediated host immunity and its evasion by *T. gondii* highlights the importance of this parasite-host interaction for the course of infection. Here, I summarize current knowledge on the roles of IFNs for the host immune reponse during *T. gondii* infection, and I highlight recent advances in understanding molecular mechanisms of IFN-mediated host defence and immune evasion. Detailed investigation of this fascinating host-parasite interaction may also identify putative target molecules that can help to develop novel parasite-directed or host-directed supportive therapeutics against toxoplasmosis.

## 2 IFN production and IFN-mediated signalling

### 2.1 IFN-α/β

**Figure 1:**
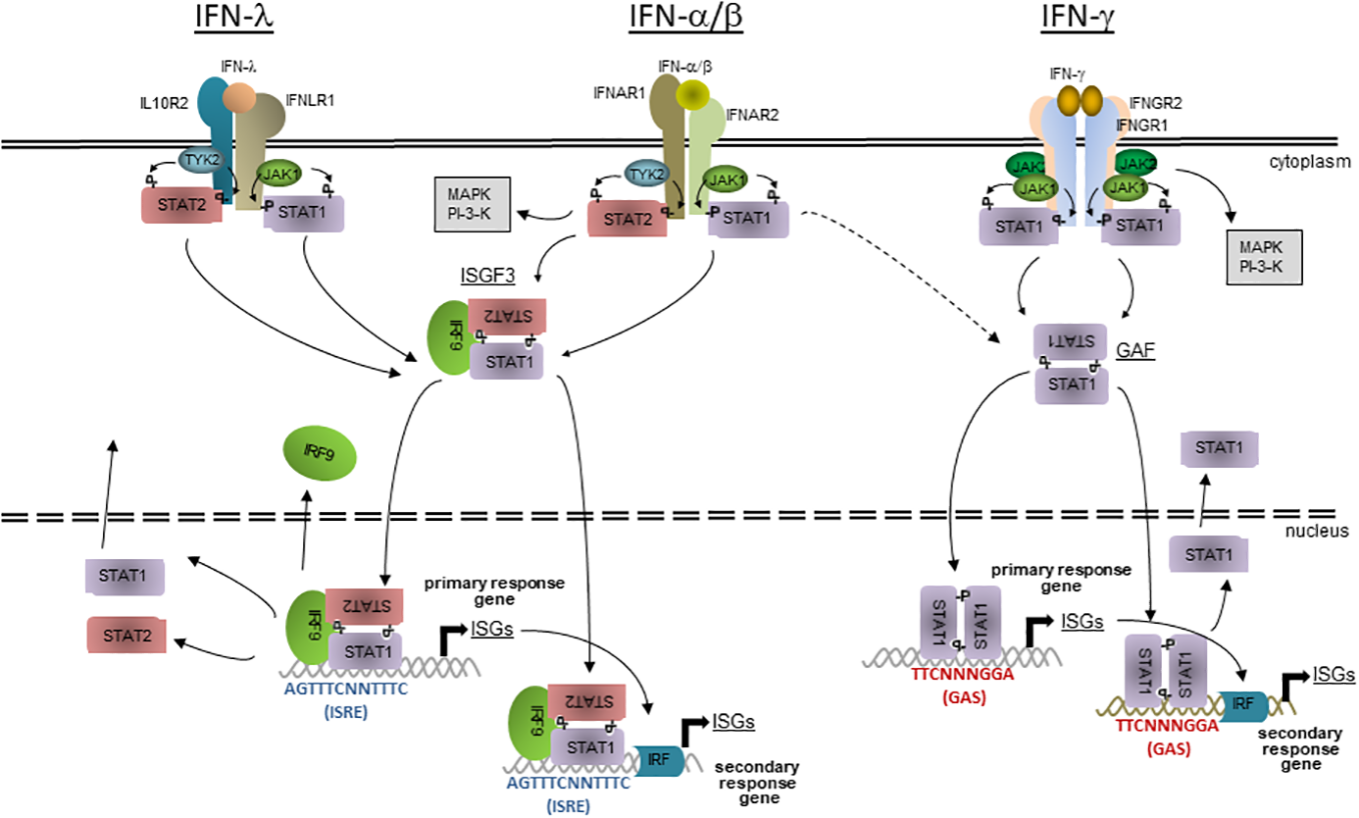
The JAK-STAT signalling of IFN-mediated gene expression. Three different classes of IFNs bind to specific cell surface receptors; IFN-α/β to IFNAR1 and 2 heterodimers, IFN-γ to IFNGR1 and 2 heterotetramers, and IFN-λ to IFNLR1 and IL10R2 heterodimers. Preassociated JAK1 and 2 (IFN-γ) or JAK1 and TYK2 (IFN-α/β and IFN-λ) activate each other and phosphorylate cytoplasmic residues on the receptor subunits, leading to the recruitment of STAT1 (IFN-γ) or STAT1 and 2 (IFN-α/β and IFN-λ). After phosphorylation, STAT1 homodimerizes (IFN-γ) or STAT1 and 2 heterodimerizes (IFN-α/β and IFN-λ), leading to fomation of GAF and, together with IRF9, of ISGF3, respectively. The active transcription factors are then imported into the nucleus, where GAF binds to GAS or ISGF3 to ISRE consensus seuquences in the promoters of primary or secondary response ISGs. Secondary response ISGs additionally require binding of transcription factors, e.g. IRFs that are expressed during the first wave of IFN-induced gene transcription. After dissociation from DNA, STATs are dephosphorylated and transported back into the cyctosol where they can eventually become reactivated at their receptors. The host responses to IFN-α/β and IFN-λ consistently overlap due to largely identical signalling modules, but is not identical due to cell type-specific expression of the specific receptors. There is also overlap between the responses to IFN-α/β and IFN-γ due to alternative formation of STAT complexes and presence of both GAS and ISRE consensus sequences in promoters of a subset of ISGs. Besides the canonical JAK-STAT signalling, IFNs can also acitvate non-canonical signalling including MAPK and PI3K cascades.

Type I IFNs can be produced by most cell types in response to stimulation by microbial PAMPs. Following oral *T. gondii* infection, the predominant source of IFN-β in mesenteric lymph nodes are inflammatory macrophages (IMs) (14). IFN-β expression by IMs requires parasite phagocytosis and recognition of *T. gondii* PAMPs in a PRR and MyD88-dependent manner rather than active parasite cell invasion (14). The role of plasmacytoid dendritic cells (pDCs), that are the main source of type I IFNs during viral infections (15, 16), for type I IFN production in response to *T. gondii* is less clear (14, 17, 18), possibly because of investigating different type I IFNs, or different host species, or different modes of parasite uptake into host cells. It is however likely that other cell types can contribute to IFN-α/β production in response to *T. gondii* (19). All type I IFNs bind – though with different affinities – to heterodimeric IFNAR1 and IFNAR2 receptor complexes that are expressed on the cell surface of most mammalian cell types. It induces autophosphorylation of pre-associated Janus kinase 1 (JAK1) and tyrosine kinase 2 (TYK2) with subsequent phosphorylation of cytoplasmic residues of IFN-αR and recruitment of signal transducer and activator of transcription (STAT) 1 and 2 (**Figure 1**). STAT1 and STAT2 are themselves tyrosine-phosphorylated, assemble together with IFN regulatory factor (IRF) 9 to form the IFN-stimulated gene factor (ISGF) 3 heterotrimer. ISGF3 is then imported into the nucleus via the importin-Ran pathway and can bind to AGTTTCNNTTTC consensus sequences (i.e., IFN-stimulated response element, ISRE) in the promoters of IFN-stimulated genes (ISGs) and activate their transcription (**Figure 1**). After dephosphorylation, STAT1 and STAT2 disassemble and are then exported back into the cytoplasm and can eventually be reactivated (20, 21). IFN-αR activation can also lead to formation of STAT homodimers including STAT1 homodimers which are normally formed in response to IFN-γ leading to partially overlapping transcriptional programmes in response to type I and type II IFNs (**Figure 1**). Activation of several non-canonical signalling pathways including MAPK and PI3K pathways in response to IFN-α/β (reviewed in (22, 23)) further expand complexity of transcription programmes in response to type I IFNs.

### 2.2 IFN-λ1-4

IFN-λs can be produced by pDCs, epithelial cells, keratinocytes, hepatocytes and primary neuronal cells after sensing microbial PAMPs (12). In an *ex vivo* toxoplasmosis model, murine intestinal organoids secreted IFN-λ2/3 after infection with *T. gondii* (preprint data in (24)), but the cellular source remains to be determined. The receptor for type III IFNs is a heterodimer composed of the IL-10R2 that is also utilized by IL-10 family members, and the high affinity IFNLR1 chain that is uniquely utilized by IFN-λs. The latter subunit is primarily expressed on epithelial cells, e.g., in the intestine or the lung, which explains the specific function of IFN-λs at mucosal surfaces. It is however also present on certain immune cells. Receptor binding initiates a similar signalling pathway as type I IFNs and induces expression of a similar but not identical set of ISGs (**Figure 1**). Different tissue distributions and differential regulation of signalling may explain emerging findings that the type I and III IFN systems differ in their impacts on infections *in vivo* (12, 25).

### 2.3 IFN-γ

In contrast to type I and III IFNs, production of type II IFN is restricted to immune cells, primarily natural killer (NK) cells, innate lymphoid cells (ILCs), T helper (T_H_) 1 and CD8^+^ cytoxic T lymphocytes (CTL) (11). Likewise, in *T. gondii*-infected mice, CD4^+^ T cells (26, 27), CD8^+^ CTLs (28–30), NK cells (31–33) and ILCs (34) produce significant amounts of IFN-γ. Neutrophils particularly contribute to early IFN-γ production at sites of acute *T. gondii* infection (35). IFN-γ binds as a homodimer to a heterotetramer of two IFNGR1 and two IFNGR2 subunits, i.e. the IFN-γR complex that is widely expressed on nearly every mammalian cell type. Close proximity of receptor-associated JAK1s and JAK2s leads to their autophosphorylation, phosphorylation of specific tyrosine residues of the IFNGR1 and recruitment of STAT1 to the receptor complex (**Figure 1**). STAT1 is then itself phosphorylated on Tyr701, dimerizes (then called gamma-activated factor; GAF) and after transport into the nucleus, binds to TTCNNNGGA consensus sequences, i.e. gamma-activated sites (GAS) in promoters of response genes (**Figure 1**). Additional phosphorylation of STAT1 at Ser727 (36) and cooperative binding of two interacting GAF dimers (37, 38) is required for full transcriptional activity of activated STAT1. After dissociation from DNA and dephosphorylation, STAT1 is exported back into the cytoplasm and can eventually be re-activated at the IFN-γR. Induction of non-canonical transcription factor complexes in response to IFN-γ, e.g. STAT1-IRF9 complexes and possibly also ISGF3 normally induced by type I IFNs can bind to ISRE-containing promoters thus leading to a partial overlapping with the canonical IFN-α/β response (39). IFN-γ can also activate several additional signalling pathways that do not require STAT1 (40).

## 3 Role of IFNs in host defence against *T. gondii*

Administration of exogenous IFN-γ protects mice against lethal *T.* gondii infection (41), whereas blocking endogenous IFN-γ (42) or genetic ablation of IFN-γ (43) or IFN-γR (44–46) abolishes parasite control and leads to host death during acute infection. Consistent with a major role of IFN-γ in resistance against *T. gondii*, STAT1-deficient mice also succumb to acute toxoplasmosis (45, 47) although these mice present defects to other IFNs as well. IFN-γ is also required to prevent the reactivation of chronic toxoplasmosis in mice (29, 48). In humans, defective IFN-γ production by T lymphocytes in response to *Toxoplasma* antigens correlates with opportunistic infections including toxoplasmosis in AIDS patients (49). Furthermore, IFN-γ-stimulated macrophages from patients with partial or complete IFNGR1 functional deficiency, but not IFN-γR-proficient controls, are unable to control *T. gondii* growth *in vitro* (50). Thus, IFN-γ is a major mediator of resistance against *T. gondii* both in humans and mice. Overproduction of IFN-γ in response of genetically susceptible mice to a high oral dose of *T. gondii* can however also mediate detrimental necrosis of mucosa of the small intestine and can lead to host death (51). Early *in vitro* studies showed that IFN-γ activates human macrophages to exert toxoplasmacidal activity (52), and this has been since then confirmed for numerous hematopoietic and non-hematopoietic cell types from different host species (e.g., (53–57)). IFN-γ hence exerts its toxoplasmacidal activity by activating cell-autonomous immunity of target host cells. It is important to note that host cells need to be activated by IFN-γ prior to infection to control or even kill the parasite, because *T. gondii* can otherwise timely prevent up-regulation of IFN-γ-induced effector mechanisms by translocating an effector molecule called *T. gondii* inhibitor of STAT1-dependent transcription (TgIST) into the host cell (see below).

IFN-α/β is also protective against *T. gondii*, although less pronounced than IFN-γ. Early studies have indicated that treatment with recombinant murine IFN-β substantially protect mice from lethal intraperitoneal infection (58). Oral infection of IFNAR1-deficient mice confirmed that type I IFN signalling contributes to host defence against *T. gondii* (14, 59). The extent of protection appears to depend on the infection dose and the resistance of mice with only modest effects of IFN-α/β during more severe courses of disease. This is supported by another study in which all mice succumbed early during acute infection with high parasite numbers regardless of presence or absence of the IFNα/βR (45). Thus, type I IFNs may protect against *T. gondii* primarily during later stages of infection, i.e. during transition towards chronic toxoplasmosis. Indeed, although *Ifnar1* ^-/-^ mice show no defect in systemic cytokine levels following parasite infection, they have higher parasite burdens in the brain and rigorous glia cell activation (59). *In vitro*, IFN-α/β diminishes parasite growth in human retinal pigment epithelial cells (60) and murine fibroblasts (61), but its effect in murine (58, 61) and human (58, 59, 62) macrophages has been inconsistent. The reason for that is not entirely clear but appears to depend on macrophage activity and co-stimulatory signals. Furthermore, type I IFN-dependent cell-autonomous parasite control is at least partially masked by the parasites’ ability to inject TgIST into the host cell thereby inhibiting IFNα/β-induced gene transcription ((59); see also below). Together, these data indicate that IFN-α/β can trigger direct anti-parasitic effector mechanisms, particularly when cells have received the IFN signal before infection. *In vivo*, type I IFNs may also contribute indirectly to host defence against *T. gondii* by regulating NK cell-mediated IFN-γ production and recruitment of inflammatory monocytes to the site of infection (59, 63).

Recently, IFN-λ was shown to partially protect mice against *T. gondii* (data available in preprint (24)). After oral infection, *Ifnlr1* ^-/-^ mice had increased parasite burdens in the ileum and succumbed earlier to infection than wildtype mice. Both intraepithelial cells (IECs) of the intestine and bone marrow-derived immune cells contribute to protection (24). Since IECs from adult mice do not efficiently respond to type I IFNs (64), IFN-λ may be particularly important and non-redundant in limiting *T. gondii* development in adult mice during initial parasite replication in the intestinal epithelium.

## 4 IFN-mediated effector mechanisms against *T. gondii* infection

A major function of IFNs is inducing effector mechanisms to combat intracellular pathogens, and several of those are effective against *T. gondii* (**Table 1**). IFNs however exert anti-parasite activity in a host species-specific and cell type-specific manner. Reactive oxygen species (ROS) can inhibit parasite growth in human and mouse professional phagocytes, and IFN-γ enhances ROS-mediated activity against *T. gondii* (**Table 1**) (52, 65, 66). Upregulation of inducible nitric oxide synthase (iNOS, NOS2) by IFN-γ together with costimulatory LPS or TNF-α and production of reactive nitrogen species (RNS) also inhibit parasite growth in murine macrophages (67–69) and skeletal muscle cells (70) (**Table 1**), but not in human macrophages (92). Importantly, RNS may also suppress lymphocyte responses to *T. gondii* during acute infection (93, 94), and they can even contribute to severe tissue damage and host death in susceptible mice (95, 96). Thus, iNOS-mediated RNS production has both pro-host and pro-parasite effects during toxoplasmosis in mice, thereby explaining that iNOS deficiency or its inhibition does not compromise parasite control during acute infection, but leads to defective immunity during chronic toxoplasmosis (68, 94, 95). In humans, induction of iNOS expression in hepatocytes even favors parasite growth by antagonizing IFN-γ-dependent indoleamine 2,3-dioxygenase 1 (IDO1) expression (97). Notably, this depends on secretion of dense granule protein (GRA) 15 by *T. gondii* which induces monocytes to secrete IL-1β and activates iNOS expression in hepatocytes in a paracrine manner (97). IDO1 depletes intracellular tryptophan that is required for intracellular growth of *T. gondii*; hence, IFN-γ-induced IDO1 upregulation and tryptophan depletion diminishes or even blocks parasite replication in various human cell types including fibroblasts and macrophages (**Table 1**) (53, 71, 72). Ectopic expression of IDO can confer toxoplasmastatic activity also to mouse rectal cancer cells (98), but endogenous IDO does not suffice to restrict *T. gondii* growth in mouse macrophages (71).

**Table 1:** IFN-regulated defence mechanisms against *T. gondii*.

Effector molecule (s)	IFN	Host species	Cell type (s)	*T. gondii* strain (s)	Mechanism of protection	References
NOXs, SOD*	IFN-γ	humans, mice	monocytes, macrophages, DCs	type I	ROS toxicity	(52, 65, 66)
iNOS	IFN-γ	mice	macrophages, muscle cells	type I, II	RNS toxicity	(67–70)
IDO1	IFN-γ IFN-β	humans	macrophages, fibroblasts, glioblastoma, endothelial cells macrophages	type I, II type I	tryptophan depletion	(53, 71, 72) (62)
IRGs, GBPs	IFN-γ	mice	macrophages, fibroblasts, astrocytes, musle cells and others	type II, III	PV disruption, parasite killing, host cell death	(55, 70, 73–80)
IRGs?* (Irgm1/Irgm3-dependent)	IFN-β IFN-λ	mice	fibroblasts, macrophages intestinal cells	type II	PV disruption, parasite growth inhibition	(61) (24)
IRGs	IFN-γ	wild-derived mice, e.g. CIM	diaphragma cells	type I, II, III	PV disruption, parasite killing, host cell death	(81)
GBP1	IFN-γ	humans	macrophages, mesenchymal stromal cells	type I, II	PV disruption, parasite killing, host cell death	(82–84)
ISG15, RNF213, ubiquitin, autophagy proteins	IFN-γ (ISG15, RNF213)	humans	epithelial cells endothelial cells, HFF	type II, III, to a lesser extent type I type II	non-canonical autophagy, parasite growth restriction PV fusion with lysosomes, acidification	(85–88) (89, 90)
Unknown	IFN-γ	humans	HFF	type I	Host cell death, premature parasite egress	(91)

*Abbreviations not previously mentioned in the main text: NOXs, NADPH oxidases; SOD, superoxide dismutase. ? denotes that a direct role of IRGs as effector molecules yet remains to be confirmed.

Not surprisingly, the PV is also an important target of IFN-γ-mediated cell-autonomous immunity to *T. gondii* in both mice and humans. It involves complex and not yet fully resolved regulatory and effector networks that considerably differ between mice and humans and between cell types in humans. Immunity-related GTPases (IRGs, formerly designated p47 GTPases) are strongly induced by IFN-γ in mice, and mice deficient in distinct IRGs are highly (*Irgm1* ^-/-^, *Irgm2* ^-/-^, *Irgm3* ^-/-^, *Irga6* ^-/-^ or *Irgb6* ^-/-^) or partially (*Irgd* ^-/-^) susceptible to *T. gondii* infection (**Table 1**) (73, 74, 99–101). After infection, IRG effectors are rapidly loaded on the PV membrane (PVM) with Irgb6 pioneering other IRGs by binding to phosphatidylinositol 5-phosphate (PI5P) and phosphatidylserine present on the PVM (76, 100). Regulatory IRGs, i.e. Irgm1, Irgm 2 and Irgm3, instead, are predominantly bound to membranes of host cell organelles but not to the PVM (102–104). According to a current model, they thereby potentially prevent IRG effector activation on ‘self’ membranes, but not on the PVM (103, 104). Notably, autophagy proteins of the microtubule-associated protein 1 light chain 3 (LC3) conjugation system are required for efficient IRG loading on the PVM although the PV is not engulfed within an autophagosome (76, 105, 106). At later time points of infection, Irgm2 and Irgm3 also localize to some PVs in activated cells, though at a smaller proportion than effector IRGs (76, 101, 107), with PVM-localized Irgm2 being required for subsequent ubiquitination (101). The PV is indeed also ubiquitinated in a manner that partially depends on p62 and the E3 ubiquitin ligases TRAF6 and TRIM21 (79, 106). This in turn allows binding of members of a second family of IFN-γ-regulated GTPases, i.e. the guanylate binding proteins (GBPs, formerly designated p65 GTPases) to the PV (75, 79). Importantly, disruption of individual or multiple GBP genes leads to increased host susceptibility towards *T. gondii* (77, 78, 80, 108). Loading of the PVM with IRGs and GBPs finally leads to disruption of the PVM, parasite death and necrotic host cell death (**Table 1**) (55, 109, 110). How the PVM is mechanistically disrupted is currently unclear. Details of requirements and timing of events of protein loading onto the PVM also need to be further refined since, for example, GBPs were shown to also regulate Irgb6 targeting to the PVM (77), i.e. a pioneer of the loading process. However, despite these open questions, IRGs and GBPs are the main IFN-γ-regulated drivers of this important defence mechanism in mice, although a complex network of other factors participates that typically functions independently of IFN-γ.

Remarkably, humans lack a functional IRG system (111), and they have evolved largely different mechanisms to target *T. gondii* at the level of the PV. Similar to mouse cells, IFN-γ triggers ubiquitination of the PVM in human epithelial cells by the E3 ligase RNF213, and leads to recruitment of autophagy proteins p62, NDP52 and LC3. This in turn, different to mouse cells, leads to formation of LC3-positive membranes around the PV and parasite growth restriction (**Table 1**) (85, 87, 88). How exactly parasite growth is compromized is unknown, but in epithelial cells it is independent of fusion with lysosomes, i.e. degradative autophagy (**Table 1**). This is different in endothelial cells, in which PVs bind ubiquitin, p62 and NDP52 but not LC3, and then fuse with lysosomes and acidify leading to parasite death (**Table 1**) (89). It should be stressed, that in these studies, only a subset of up to 30% of PVs became positive for the different markers, and that the different PV targeting pathways in endothelial and epithelial cells are obviously not mutually exclusive but may rather represent predominant fates (85, 89). Recently, IFN-stimulated gene (ISG) 15 was identified as the link that connects IFN-γ-dependent gene expression and the core autophagy machinery, possibly by binding to and targeting p62 to the PVM (86). Yet another mechanism exerts anti-*T. gondii* activity in human macrophages and mesenchymal stromal cells. It involves recruitment of GBP1 to the PVM, with its subsequent lysis and parasite killing (**Table 1**) (82–84). Recruitment of GBP1 to the PVM is facilitated by depletion of the IFN-γ-regulated kinase PIM1 in infected macrophages thereby restraining GBP1 phosphorylation and sequestration (112). At least in macrophages, this leads to recognition of parasite DNA by AIM2 and caspase 8-dependent apoptosis-like host cell death (83). Whether GBP1 targeting to the PVM depends on prior ubiquitination and recruitment of core autophagy proteins is an important questions that needs to be investigated in the future. In other human cell types, GBPs did either not restrict *T. gondii* growth (82, 91, 113) or limited parasite growth by yet unknown mechanisms independently of the PV (114, 115). In human foreskin fibroblasts (HFF), IFN-γ triggers host cell death that correlates with premature parasite egress thereby limiting parasite propagation (**Table 1**) (91). Thus, in humans, IFN-γ can activate diverse parasiticidal programs that significantly differ between cell types and that extensively deviate from the IRG/GBP-dependent defence mechanism operating in mice.

The effector molecules that restrict *T. gondii* growth in response to IFN-α/β are less well studied, but the overlapping transcriptional responses to type I and type II IFNs suggest also partial overlap of effector mechanisms. IDO-induced tryptophan deprivation and Irgm1-dependent loss of PV integrity have been implicated to control *T. gondii* in human macrophages and murine hematopoietic and non-hematopoietic cells, respectively (**Table 1**) (61, 62), similar to what has been shown in response to IFN-γ. Unexpectedly however, Irgm1 was supposed to localize to the *T. gondii* PVM prior to its disruption (61), a finding that contradicts the predominant binding of Irgm1 to host cell organelle membranes following IFN-γ stimulation (102, 103). Disruption of the *T. gondii* PV was also not confirmed in diverse human and mouse cell types after infection with a parasite mutant that is unable to inhibit type I IFN-induced transcription (i.e. ΔTgIST; see also below (59)). Thus, the role of the IRG/GBP system as an effector mechanism in IFN-α/β responses in murine cells needs to be further explored. *In vivo*, type I IFNs contribute also indirectly to host defence against *T. gondii* by stimulating NK cells to secrete IFN-γ and recruitment of inflammatory monocytes to the site of acute infection (59).

Partial protection of mice by IFN-λ appears to depend on the IRG system since IFN-λ-mediated inhibtion of parasite growth is abolished in intestine organoid-derived cells from *Irgm1^-/-^/m3^-/-^* double knockout mice as compared to wildtype mice (**Table 1**; data in preprint (24)). Absence of both Irgm1 and Irgm3 leads to mistargeting of effector IRGs and prevents efficient loading of IRGs and GBPs to *T. gondii* PVs (103). This suggests that ineffecient loading of the PVMs with IRGs/GBPs in *Irgm1/m3^-/-^* intestinal cells can be responsible for the lack of parasite inhibition in response to IFN-λ. However, Irgm proteins can also regulate autophagy, and IFN-λ treatment of intestinal cells efficiently triggers loading of the PVM with Irgb6 only, but not Irga6 and Irgb10 (data in preprint (24)). Thus, the role of the IRG/GBP system in IFN-λ-induced defence against *T. gondii* in the intestinal epithelium needs to be further confirmed.

## 5 *Toxoplasma gondii* strain-specific differences in susceptibility to IFNs

In Europe and North America, three clonal parasite lineages predominate, i.e. type I, II and III, while in South America, *T. gondii* is genetically more diverse (116). Strains largely differ in their virulence, and this has been extensively studied in mice, i.e. natural hosts of the parasite. Type I strains are highly virulent in laboratory mice, while type II and III strains are less virulent (117, 118). South American strains are generally considered virulent to mice and humans, which may account for the high disease burden imposed by *T. gondii* in that region (119). Importantly, polymorphic proteins that are secreted from the rhoptry (ROP) and dense granule (GRA) organelles into the host cell are decisive for virulence traits in mice by regulating host signalling pathways including IFN-triggered host effector functions (120–123).

**Figure 2:**
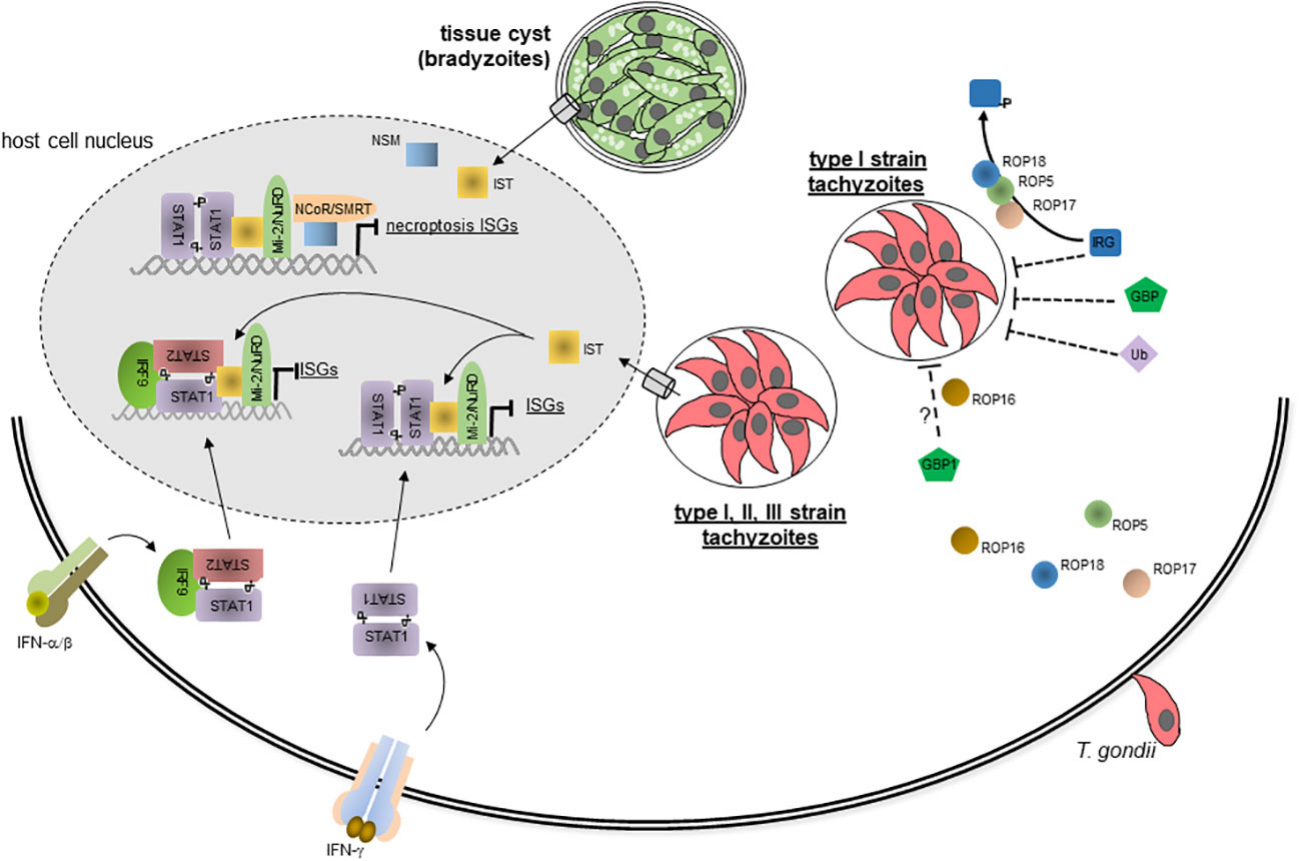
Evasion of IFN-mediated immune responses by *T. gondii*. During host cell invasion, *T. gondii* secretes several effector proteins including ROP5, ROP16, ROP17 and ROP18 from the rhoptries into the host cell where they can inhibit loading of the PVM with IRGs, GBPs and Ub in a *T. gondii* strain-specific manner. This largely abolishes disruption of the PVM containing type I strain parasites and subsequent parasite and host cell death. Parasites of the three canonical type I, II and III strains also secrete dense granule protein IST in a translocon-dependent manner into the host cell, where it traffics into the host cell nucleus. By binding to active STAT1 dimers, i.e. GAF, or to STAT1-STAT2-IRF9 complexes, i.e. ISGF3, IST prevents IFN-γ and IFN-α/β triggered chromatin remodelling and recruitment of components of the basal transcriptional mashinery thereby abolishing global expression of IFN-induced ISGs. IST also recruits the repressor complex Mi-2NuRD to ISG promoters although this appears to be dispensable for inhibiting IFN-induced gene transcription. Bradyzoite-containing tissue cysts of *T. gondii* secrete the dense granule protein NSM into the host cell, where it also traffics to the host cell nucleus, recruits the NCoRSMRT repressor and synergizes with IST to inhibit necroptosis-regulating ISGs. ? denotes that mechanisms of ROP16-mediated inhibition of GBP1 recruitment are yet unclear.

With regard to IFN-mediated host responses, loading of PVMs with IRGs and GBPs and subsequent vacuole lysis is strongly reduced after infection with type I as compared to type II and III parasites (75, 76, 124, 125). Genetic crosses between different parasite strains and subsequent functional analyses initially identified the serine-threonine kinase ROP18 and a family of closely related ROP5 pseudokinases differentially regulating IRG loading (**Figure 2**) (121, 126–132). ROP5 has the largest impact on virulence by enhancing ROP18-mediated phosphorylation of Irga6 and by blocking Irga6 oligomerization, both contributing to inactivation of Irga6 (130, 131). ROP17 is another kinase that interacts with ROP5 and preferentially phosphorylates and inactivates Irgb6 (**Figure 2**) (133). GRA7, GRA60 and ROP39 also associate with ROP18 and/or ROP5 and likely form a multi-subunit complex on the cytosolic side of the PVM and synergize to inactivate the IRG system (134–137). However, only polymorphic ROP5 and ROP18 determine parasite virulence by differentially activating ROP18 and blocking Irga6 or by differences in expression levels, respectively. Remarkably, mouse IRG genes are also highly polymorphic, and distinct haplotypes present in wild-derived mice confer resistance against otherwise highly virulent *T. gondii* strains by putative binding to ROP5 and allowing IRG loading on the PVM (**Table 1**) (81). This indicates evolutionary co-adaptation of the mouse IRG system and *T. gondii* virulence effectors and highlights the importance of IFN-regulated IRGs for the host-pathogen interaction in mice. Since IRGs and GBPs are loaded on PVs in a coordinated fashion, it is not surprising that ROP5 and ROP18 also differentially regulate GBP binding to the PVM depending on parasite virulence (**Figure 2**) (78, 125). Likewise, ubiquitination of PVMs surrounding virulent type I strains is also inhibited in a ROP18-dependent fashion (**Figure 2**) (79). ROP16 of type I parasites and GRA15 of type II parasites additionally inhibit or promote, respectivly, GBP1 and ubiquitin loading of the PVM (**Figure 2**) (79, 125). ROP16_type I_ and GRA15_type II_ can directly phosphorylate STAT3/6 and activate NF-κB in a strain-specific manner, and they contribute to *T. gondii* virulence (13). With respect to the IRG/GBP-mediated immune defence, GRA15_typeII_ is able to bind the TRAF6 ubiquitin ligase (see above) and thereby recruits p62, LC3, IRGs and GBPs specifically to PVs of *T. gondii* type II parasites, at least within murine fibroblasts (90). It consequently promotes IRG/GBP-dependent lysis of type II parasite PVs, indicating that positive regulatory mechanisms also contribute to strain-specific parasite susceptibility to the IRG/GBP-mediated immune effector function in mice. How ROP16_typeI_ regulates protein recruitment to the PVM mechanistically is unknown. The ROP54 pseudokinase has been shown to inhibit GBP2 but not Irgb6 loading on PVs of type II parasites (138), indicating that GBPs can be specifically targeted by distinct parasite effectors. Together, these results highlight the critical impact that evasion of the IRG/GBP system has on parasite virulence in mice.

In human cells, ROP5 and ROP18 are not relevant for parasite survival in IFN-γ-primed fibroblasts (132, 139), consistent with the IRG/GBP system being dispensible for parasite control. Parasite strains nevertheless differ in their susceptibility to IFN-γ-triggered ubiquitination and loading with core autophagy proteins of the PVM in epithelial and endothelial cells, with type I parasites being again considerably more resistant as compared to type II and III parasites (85, 89). Importantly, increased loading of PVs containing type II and III parasites with the ubiquitin-autophagy protein system leads to more vigorous growth restriction of these parasites than type I parasites (85). It is important to note however, that type I parasites within the small number of PVs that are targeted by the non-canonical ubiquitin-autophagy pathway are also growth-restricted. Thus, the major difference between the parasite strains at least in human epithelial cells appears to be the percentage of PVs recruiting these host proteins and the level of recruitment in response to IFN-γ (85). Mechanistically, and similar to mouse fibroblasts (see above), GRA15 from type II *T. gondii* strains increases IFN-γ-triggered recruitment of p62, NDP52 and LC3 to the PVM in human fibroblasts by binding TRAF2 and TRAF6 (90). Due to the different downstream effector mechanisms of these pathways in mice and humans, in human fibroblasts, this however rather facilitates fusion of type II strain PVs with lysosomes and subsequent parasite killing and growth restriction. Additional parasite effectors that regulate the differential resistance and susceptibility traits in human cells remain being identified.

Differences in the regulation of IFN responses also applies to a wider range of parasite strains. Expression of transcription factors involved in type I IFN induction and responses including STAT1, IRF1, IRF2, IRF7, IFN-β and IFN-inducible proteins significantly differ between *T. gondii* strains of global diversity, with distinct atypical strains specifically inducing high levels of IFN-β in murine macrophages (140). Mechanistically, increased intracellular killing of these parasites and recognition of parasite PAMPs has been proposed to increase the type I IFN response. Strain-specific activation of IFN-β and downstream effectors also apply to human cells (140).

## 6 Evasion of IFN-mediated immunity by *T. gondii*

As discussed in the previous chapter, IFN-mediated growth restriction or even killing of *T. gondii* can be abolished by type I parasites by counteracting the recruitment to the PVM of IRGs/GBPs in mice or the ubiquitin-autophagy system in distinct human cells (**Figure 2**). Yet another mechanism of *T. gondii* to counteract the IFN-mediated immunity is the inhibition of IFN-regulated gene expression (**Figure 2**). It relies on secretion of the dense granule protein *T. gondii* inhibitor of STAT1-dependent transcription (TgIST; see also above) into the host cell where it accumulates in the host cell nucleus via recognition of putative nuclear localization sequences (141, 142). Importantly, TgIST interacts with GAF, i.e. activated STAT1 dimers, thereby globally blocking IFN-γ-dependent gene expression (**Figure 2**) (143, 144), including critical mechanisms of immunity, e.g. major histocompatibility complex expression, antigen presentation to T cells or iNOS-mediated RNS production (69, 145, 146). IFN-γ-mediated gene expression is abolished in various hematopoeietic and non-hematopoeietic cell types from humans and mice (142, 143, 145, 147, 148). Furthermore, TgIST is conserved among various parasite strains and enables all three canonical type I, II, and III strains to inhibit IFN-γ-regulated gene expression (142, 149). Hence, inhibition of IFN-γ-regulated gene expression is a broadly acting and general immune evasion mechanism of *T. gondii*, suggesting its importance for the pathogenesis of toxoplasmosis. Indeed, deletion of *Tgist* renders parasites avirulent and enables mice to control the infection in an IFN-γ-dependent manner (141, 142). Mechanistically, TgIST binds via internal repeat regions to STAT1 dimers (**Figure 2**) and thereby sterically hinders STAT1 interaction with the histone acetyltransferase coactivator CBP/p300 (150) and possibly recruitment of other components of the basal transcriptional machinery including the nucleosome remodeling complex BAF (Brahma-related gene (BRG)/Brahma (BRM)-associated factor) (144). Mutational analyses confirmed that binding of TgIST via the internal repeats to STAT1 suffices to block the transcription of primary and secondary IFN-γ response genes (150). IFN-γ-triggered chromatin remodeling including histone post-translational modifications are consequently broadly abolished in infected cells (142, 144, 151). TgIST furthermore stabilizes interaction of STAT1 with GAS-containing promoters (141, 152, 153). At first glance, this appears counterintuitive, but since STAT1-TgIST complexes do not lead to promoter activation and ongoing transcription, it rather sequesters STAT1 at inactive promoters thereby reducing STAT1 dephosphorylation, nuclear export and eventually STAT1 reactivation at the IFN-γ receptor. Finally, *T. gondii*-infection facilitates binding of activated STAT1 complexes even to non-GAS DNA sites further contributing to aberrant sequestration of STAT1 (153). Whether the latter effect is mediated by TgIST still needs to be confirmed. Remarkably, TgIST also binds and recruits the chromatin repressor and deacetylase complex Mi-2/NuRD to GAS-containing promoters (**Figure 2**) (141, 142); however, this appears to be dispensable for repression of IFN-γ-regulated gene expression and for parasite virulence *in vivo* (150). Hence, TgIST largely blocks STAT1-dependent gene expression by interfering with chromatin remodelling, and the epigenetic landscape indeed crititcally impacts the ability to control IFN responses (151). Besides IFN-γ-mediated gene expression, TgIST also blocks IFN-β-mediated gene expression by binding to ISGF3 (i.e., STAT1-STAT2-IRF9), recruitment of Mi-2/NuRD and sustained binding of ISGF3 to DNA (**Figure 2**) (59, 152). Molecular mechanisms are supposedly similar to those described for inhibiting IFN-γ responses, although a more prominent role of Mi-2/NuRD recruitment to type I IFN-responsive promoters can not be excluded. The impact of TgIST on IFN-λ-triggered gene expressions has not yet been investigated, but due to the similar signalling as observed in response to IFN-α/β, it may also block gene expression in response to these IFNs.

Another *T. gondii* effector molecule that inhibits type I and type II IFN responses is *T. gondii* NCoR/SMRT modulator (TgNSM) (154). TgNSM is a dense granule protein that also translocates to the host cell nucleus where it increases the level of NCoR/SMRT repressor complex and represses IFN-regulated genes related to a form of programmed necrosis, i.e. necroptosis (**Figure 2**). It synergizes with TgIST to inhibit IFN-γ-induced necroptosis during the chronic stage of infection thereby significantly increasing survival of the latent tissue cysts (154).

Recently, several CRISPR/Cas9 screens were conducted in order to identify additional *T. gondii* proteins that are required to counterbalance IFN-γ-mediated immune responses and/or determine parasite fitness *in vivo* (139, 155–157). Among the identified proteins was ROP1 which contributes to *T. gondii* survival in IFN-γ-stimulated murine and human macrophages and to the virulence of type II strains *in vivo* (156). ROP1 is secreted by *T. gondii* during host cell invasion, localizes to the PVM, and interacts with several host proteins; however, molecular mechanisms of ROP1 to counteract IFN-γ host responses are still unknown (156). Several previously unrecognized GRA proteins were also identified in these screens, but mechanisms how they confer parasite resistence towards IFN-γ-activated cells either remain unknown (139, 157), or they are required for localization and secretion of other GRA effectors rather than directly mediating immune evasion (155).

## 7 Regulation of host IFN responses to *Toxoplasma*

The essential role of IFNs for combating *T. gondii*, and the parasite’s ability to subvert IFN-mediated host defence has prompted research into how to foster IFN host responses to restrict *T. gondii* more effectively. For example, a genome-wide mammalian cDNA library screen identified several enhancers of STAT1-dependent responses which increase IFN-γ-mediated host responses to *T. gondii* infection (158). Among them, the orphan nuclear receptor TLX enhances expression of a subset of IFN-γ-regulated genes by its DNA-binding activity and by increasing STAT1 binding to GAS-containing promoters. Interestingly, TLX is upregulated in numerous cells in the brains of *Toxoplasma*-infected mice, and it restricts parasite burden in the brain during chronic toxoplasmosis (158). Whether TLX or one of the other STAT1 enhancers identified in that screen can be therapeutically targeted to potentiate toxoplasmocidal activities remains to be explored in the future. Since *T. gondii* blocks histone modifications including acetylation at STAT1-responsive promoters (see above; (142, 144)), we reasoned that increasing histone acetylation may rescue infected cells from parasite-induced inhibition of IFN-γ responses. Treatment of infected cells with histone deacetylase inhibitors indeed abrogated parasite-induced blocking of histone acetylation at promoters of distinct IFN-γ secondary response genes and potentiated MHC class II expression in infected cells (144). However, a subsequent genome-wide transcriptome analyses showed that the HDAC inhibitor MS-275 does not generally restore the defective responsiveness of *T. gondii*-infected macrophages to IFN-γ at the transcriptional level and does not augment anti-parasite host defence (159). Using an untargeted compound library approach, Radke et al. identified several compounds which specifically synergized with IFN-γ to augment anti-parasitic host responses in human cells (160). Several of the hits increased recruitment of LC3 to the PV (see above), suggesting a more effective autophagy-related defence mechanism towards *T. gondii* (160). Together, these studies highlight the potential of targeting IFN-regulated host responses, or alternatively, parasite-mediated evasion of them to develop supportive therapies against toxoplasmosis in the future.

## 8 Conclusions and perspectives

IFN-γ and to a lesser extent also type I and III IFNs are major mediators of host resistance against *T. gondii*. The parasite on the other hand has evolved efficient means to counteract IFN-mediated immune defence. The balanced interplay between host IFN responses to infection and parasite effectors mediating evasion of IFN-regulated transcription and effector functions have emerged as being decisive to control parasite replication and to facilitate parasite persistence and hence transmission to new hosts.

As discussed above, considerable progress has been made during recent years in understanding the molecular mechanisms that mediate host defence against *T. gondii* and those mechanisms *T. gondii* has evolved to counteract host IFN responses. The identification of an arsenal of parasite ROPs and GRAs that inactivate the IRG/GBP loading of the PVM in mice or that even abolish the IFN-induced transcriptional mashinery highlights the importance of these immune evasion mechanisms for the infection biology of *T. gondii*. Despite this progress, there are however several important issues that still needs being resolved. For example, how is loading of the PVM by IRGs, GBPs and accessory proteins spatially and temporally regulated, and how is the PVM mechanistically lysed? Likewise, how do the different cell type-specific anti-parasitic effector mechanisms that target the PVM in human cells contribute to the overall host defence *in vivo* and how is that precisely regulated? What *T. gondii* effectors compromize PVM loading with ubiquitin/autophagy proteins in type I strain-infected human cells? What is the exact contribution of reduced histone modification, STAT1 sequestration or inhibition of STAT recycling in abolishing IFN-induced gene transcription by TgIST? And finally, what is the role of Mi2/NuRD recruitment by TgIST in inhibiting IFN-induced gene expression, and how is IFN-triggered gene repression abolished by *T. gondii*? Answering these questions will not only further understanding of fascinating and critical host parasite interactions. It will also help identifying novel factors and mechanims that regulate IFN responses against intracellular pathogens in general and potentially microbial effectors subverting them. It may also lead to identification of potentially promising target molecules the modulation of which can foster anti-parasitic host responses thereby supporting effective anti-parasitic therapy. Examples of such regulators of IFN responses as well as chemical compounds that are able to augment IFN-γ responses have indeed been identified during recent years (158, 160). It will be interesting to see whether and how this information can be translated into novel options for the effective treatment of toxoplasmosis in the future.

## Author contributions

CL: Conceptualization, Funding acquisition, Visualization, Writing – original draft, Writing – review & editing.
